# Virus transmission frequencies in the pine root rot pathogen *Heterobasidion annosum*

**DOI:** 10.1016/j.virusres.2024.199467

**Published:** 2024-10-07

**Authors:** Elina Roininen, Eeva Johanna Vainio, Suvi Sutela, Anna Poimala, Muhammad Kashif, Tuula Piri, Jarkko Hantula

**Affiliations:** a University of Helsinki, Viikinkaari 1, Helsinki FI-00790, Finland; b Natural Resources Institute Finland (Luke), Latokartanonkaari 9, Helsinki FI-00790, Finland

**Keywords:** *Heterobasidion*, Transmission, Partitivirus, Ourmiavirus, Mitovirus, Co-infection

## Abstract

The combined use of Heterobasidion partitiviruses 13 and 15 (HetPV13-an1 and HetPV15-pa1) is considered a promising biocontrol approach against Heterobasidion root and butt rot. In a previous study, the transmission frequency of HetPV15-pa1 was found to be higher from a double partitivirus-infected donor than from a single partitivirus-infected donor. In this study, we included a wider array of recipient isolates to assess whether the phenomenon is widespread across different host strains and conducted transmission experiments on artificial media (*in vitro*) using a total of 45 different *H. annosum* donor-recipient pairs. In addition to investigating whether double partitivirus infection improves the transmission of HetPV13-an1 and HetPV15-pa1, we examined for the first time how efficiently co-infecting ssRNA viruses are concomitantly transmitted with the partitiviruses, and whether pre-existing ssRNA viruses in the recipients affect virus transmission. Generally, the transmission rates of HetPV13-an1 and HetPV15-pa1 were high from both single partitivirus-infected and double partitivirus-infected donors to most of the *H. annosum* recipient strains, with few exceptions. However, in contrast to previous experiments, the transmission frequency was not higher from the double partitivirus-infected donors. Also, ourmiavirus was transmitted between *H. annosum* strains, but the presence of another ourmiavirus in the recipient might affect the efficacy.

## Highlights

• High frequency in transmissions of Heterobasidion partitiviruses 13 and 15.
• Recipient extant infections did not inhibit the transmission.
• Mitovirus transmission was rare between *H. annosum* strains.
• Ourmiavirus transmission was common in coinfection with mito- and partitiviruses.

## 1 Introduction

*Heterobasidion annosum* (Fr.) Bref. *sensu stricto* is a widespread pathogen of conifers and it is responsible for a disease called Heterobasidion root rot (Niemelä and Korhonen, 1998)*.* Primary infection happens by airborne basidiospores that settle on fresh stumps (Redfern and Stenlid, 1998). The fungus then colonizes the root system and spreads to nearby trees through connected roots (Korhonen, 1978; Redfern and Stenlid, 1998; Woodward et al., 1998).

The diverse community of mycoviruses with double-stranded RNA (dsRNA) or single-stranded RNA (ssRNA) genomes has been recently revealed (Kondo et al., 2022; Ayllón and Vainio, 2023; Sato and Suzuki, 2023). They are commonly found in various fungal species, but typically do not produce infectious particles outside the cell. As a result, they move between cells through hyphal connections called anastomoses or via sexual or asexual spores (Hillman and Milgroom, 2021; Ayllon and Vainio, 2023; Hough et al., 2023). However, anastomosis is short-term between species and vegetatively incompatible strains of the same species, which restricts viral transmission. The mycoviruses infecting *H. annosum* species are classified within or related to classified members of the families *Partitiviridae, Curvulaviridae, Mitoviridae, Narnaviridae, Botourmiaviridae, Dumbiviridae, Trimbiviridae and Fusariviridae* (Kuhn et al., 2024; Dálya et al., 2024).

Most of the mycoviruses do not cause phenotypic changes, but some of them have debilitating or hypovirulence effects (Day et al., 1977; Ahn and Lee, 2001; Osaki et al., 2002; Yu et al., 2010; Vainio et al., 2018), which has raised interest in using them as biocontrol agents (Ghabrial et al., 2015; Vainio and Hantula, 2016; van Diepeningen 2021; Hough et al., 2023; Vainio et al., 2024). Vainio et al. (2018) and Kashif et al. (2019) showed that two viruses, Heterobasidion partitivirus 13 strain an1 and Heterobasidion partitivirus 15 strain pa1 (HetPV13-an1 and HetPV15-pa1), of *Partitiviridae* family, *Alphapartitivirus* genus, caused growth reduction and negative phenotypic effects on certain *H. annosum* strains. However, some *H. annosum* strains have shown tolerance to these effects, and other viruses may influence their phenotypic effects and transmission frequencies between two mycelia *in vitro* (Kashif et al., 2019; Hantula et al., 2020).

Partitiviruses are known to spread relatively efficiently between vegetatively incompatible *Heterobasidion* sp. strains (Vainio and Hantula, 2016), but the spread and co-infection effects of many virus species, like mitoviruses and ourmiaviruses, has not been investigated extensively in *H. annosum*. The virus transmission efficiency, phenotypic effects, or interactions of ssRNA viruses with dsRNA viruses in co-infections of partiti-, ourmia- and mitoviruses are not known in *H. annosum.* In this investigation our aim was to elucidate the feasibility of simultaneous use of two biocontrol viruses by examining whether co-infecting HetPV13-an1 and HetPV15-pa1 enhance each other's transmission rates between the majority of *H. annosum* isolates, as Kashif et al. (2019) hypothesized. We also recorded whether pre-existing virus infections by other viruses in the recipient affect the transmission of partitiviruses, and how efficiently members of *Mitoviridae* (Heterobasidion mitovirus 1 (HetMV1-an1), Heterobasidion mitovirus 2 (HetMV2-an1) and Heterobasidion mitovirus 3 (HetMV3-an1); genus *Mitovirus*) and *Botourmiaviridae* (Heterobasidion ourmia-like virus 4 (HetOlV4-an1)) are transmitted from isolates co-infected by partitiviruses.

## 2 Materials and methods

### 2.1 RNA sequencing

RNA sequencing was done for two pools of samples (Supplementary Table 1). Elina01 library comprised ten *H. annosum* isolates, while Elina02 included seven *H. annosum* isolates and three additional basidiomycete isolates (not included in the present study: *Lactarius rufus, H. abietinum, Paxillus* sp.). RNA sequencing (RNA-seq) was done to detect RNA viruses and actively transcribed DNA viruses in the host fungi. Strains were cultivated on modified orange serum (MOS) agar plates (3 % w/v Orange Serum Agar (HiMedia Laboratories, Maharashtra, India), 0.8 % w/v Bacto™Malt Extract (Detroit, MI, USA), 0.8 % w/v d-glucose anhydrous (VWR, Solon, Ohio) and 0.3 % w/v Agar (VWR, Solon, Ohio, USA)) at +20°C for 14 days. The grown mycelium was collected and freeze-dried for one to two days before storage at −80°C. Samples were homogenized using FastPrep-24 (MP Biomedicals, Irvine, CA, USA), and RNA was extracted with the Spectrum Plant Total RNA Kit (Sigma-Aldrich, Darmstadt, Germany) as per the manufacturer's instructions. RNA quantity was determined using NanoDrop (Thermo Fisher Scientific, Madison, WI, USA), and quality was assessed via agarose gel electrophoresis. Samples were divided into two pooled libraries Elina01 and Elina02, each containing 1 μg of RNA from ten isolates. RNA-Seq was performed on rRNA-depleted total RNA, with further quality checks, library construction, and sequencing conducted at facilities of Macrogen Europe. RNA integrity numbers (RINs) were 8.5 for Elina01 and 7.3 for Elina02. Libraries were constructed using TruSeq Stranded Total RNA with Ribo-Zero H/M/R Gold (Illumina, San Diego, CA, USA), and sequencing employed an Illumina NovaSeq 6000 system, generating stranded paired-end sequences.

Bioinformatic analysis of RNA-seq data was adapted from the pipeline of Sutela et al. (2021). The reads were cleaned using Trimmomatic and *de novo* assembled with Trinity (v. 2.8.5). BLASTx (v. 2.10.0, evalue 10e-6) with predicted proteins of hosts (*H. irregulare)* (Hannosum_v2.FilteredModels1.proteins.fasta; Olson et al., 2012), *Paxillus involutus* (Kohler et al., 2015), and *Lactarius* sp. (Lebreton et al., 2022)) were utilized in cleaning the host originated Trinity contigs out. The host-depleted contigs were compared (BLASTx, v. 2.10.0, evalue 10e-5) to a custom viral database (containing proteins of *Riboviria, Genomoviridae*, and unclassified viruses retrieved from NCBI with some filtering of most abundant viral sequences). Virus-like sequences detected in each RNA-Seq library were allocated to specific fungal isolates by RT-PCR using virus specific primers (Supplementary Table 2). When more than one strain hosted isolates of the same virus species, we Sanger sequenced the RT-PCR products at Macrogen Europe (Amsterdam). Mapping of raw reads was done against the Trinity contigs with Geneious Prime using default settings and low sensitivity option, and single nucleotide polymorphisms (SNPs) detected using default settings and 0.15 minimum variant frequency. When only one strain in the sequencing library hosted a specific virus species, the corresponding Trinity contig was submitted to GenBank as a metagenomically assembled genome (MAG). Instead, when there were multiple virus variants of the same virus species, we submitted the Sanger sequenced RT-PCR products (Poimala and Vainio, 2023).

### 2.2 Heat treatments

Ten *H. annosum* strains (pool Elina01; Supplementary Table 1) were subjected to heat treatment by first growing the mycelium on 2 % malt extract agar plates (MEA; 2 % w/v Bacto™Malt Extract, Detroit, MI, USA and 1.5 % w/v Agar, VWR, Solon, Ohio, USA) at 20°C for two days, and thereafter two days at 28°C, one week at 30°C and finally, one week at 33°C. This was followed by three days recovery of the fungal mycelia on the heat-treated plates at room temperature and then transferring an inoculum of the newly growing mycelium to fresh MEA plates to further recovery that was visually assessed and appeared to be normal in growth rate and morphology. After two months of recovery, the success of heat-treatment in removing of the extant virus infections was tested by RT-PCR with virus specific primers. A set of 6 *H. annosum* recipients was used in transmission experiments without heat treatment, each hosting 1 to 6 pre-existing viruses. The strains of the pool Elina02 were not heat-treated (Supplementary Table 1).

### 2.3 Virus transmissions

Virus transmission was examined in three different experiments. The first experiment used *H. annosum* 94233 strains hosting either HetPV13-an1 or HetPV15-pa1 and co-infecting mito- and ourmiaviruses as donors and three virus-free and three virus-infected recipients (Table S1). In the second experiment, the same recipients were used but the donors were *H. annosum* 03021 strains hosting HetPV13-an1 with an ourmiavirus or HetPV13-an1 with HetPV15-pa1 and an ourmiavirus. Finally, the third experiment tested virus transmission from *H. annosum* 94233 strains hosting both of the two partitiviruses or each partitivirus alone (all cases with co-infecting mito- and ourmiaviruses) into seven heat-treated recipients with variable viral content (Table S1). Also, the strains Hausjärvi 4.2 and Lokalahti 1.7 (listed in Supplementary Table 1) were initially recipients of the third experiment, but the pairing tests revealed mixing of recipient and donor hyphae observed during processing the samples after dual culture (data not shown).

Incubation of dual cultures of virus donors and recipients was conducted on MEA plates using five replicates of each donor/recipient pair. Donors of the strain 94233 grew slower and were incubated 14 days at 20°C before the recipient strain was inoculated on the same plate. The inoculum was an approximately 0.5 × 0.5 cm piece of MEA containing actively growing mycelia taken about 1 cm from the edge of the culture. The inoculant pieces were placed on the opposite sides of the plate about 1 cm from its edge. After 60 days incubation at 20°C, subcultures were made by cutting three pieces of size 0.5 × 0.5 cm from the recipient side approximately 1.5 to 2 cm from a demarcation zone between the (vegetatively incompatible) mycelial colonies and placed on cellophane covered plates.

Mycelia were harvested from the plates, and approximately 50 mg (wet weight) was used for total nucleic acid extraction. Nucleic acids for RT-PCR were extracted using a protocol based on chloroform-phenol extraction, PEG precipitation and ethanol wash as described in Vainio et al. (1998). cDNA was produced using approximately 2 μg of total nucleic acids and RevertAid Reverse Transcriptase (Thermo Scientific, Vilnius, Lithuania) according to manufacturer´s instructions, except that Ribolock was not used, the amount of RevertAid was 0.5 μl and the incubation time at 42°C was two hours. RT-PCR for detection of transmitted viruses was done as described by the polymerase manufacturer using virus specific primers (Supplementary Table 1) and DreamTaq DNA Polymerase (Thermo Scientific, Vilnius Lithuania). PCR products were detected with agarose gel electrophoresis and visualized by UV light using ethidium bromide stain (VWR Chemicals, Solon, Ohio, USA).

Vegetatively incompatible strains of *H. annosum* generally form a demarcation zone as a visible mark of the rejection by each other (Stenlid, 1985). Nevertheless, the strength of this reaction varies and sometimes the two mycelia might be intermingled. To eliminate possible presence of the donor strain at the recipient side, positive subcultures were purified thrice from the very edge of the 2 to 4 days old mycelium to obtain pure cultures. The presence of viruses was tested again as described above by RT-PCR with virus specific primers (Supplementary Table 2). Then pairing tests were made for the virus hosting subcultures against donor and original recipient strains (Stenlid, 1985). Further recipient genotyping was conducted using random amplified microsatellite (RAMS) profiling (Hantula et al., 1996) in the case of the recipient Köyliö K2 and donor 94233-PV13-MV1-2 using DreamTaq DNA Polymerase and CT primers (Supplementary Table 2). Fisher's exact test was used to calculate p-values when comparing the transmission frequencies between specific donor-recipient combinations.

## 3 Results

### 3.1 Viruses detected by rna sequencing

The natural mycovirus infections of *H. annosum* strains utilized in the transmission experiments were examined with RNA-seq followed by RT-PCR. The raw reads of the libraries Elina01 (SRR29430553) and Elina02 (SRR29430552) are available at NCBI under Bioproject ID PRJNA1105565. The analysis conducted before heat-treatment showed that the isolates were often infected by one or more viruses (Supplementary Tables 1 and 3). Viral content of the isolates used in the transmission experiments is reported in Supplementary Table 1. Briefly, the isolates hosted earlier characterized and new partiti- curvula-, ourmia-, narna- and ambiviruses (accessions OR343711-OR343738), whose affiliation with known viruses based on BlastX analysis is reported in Supplementary Table 4 (more detailed phylogenetic analysis of the viruses is beyond the scope of this study).

Notably, HetOlV4-an1 was detected by RNA-Seq from the *H. annosum* donor 03021-PV13-15-OlV4 and by RT-PCR from other *H. annosum* donor strains (Supplementary Table 1). This virus had remained undetected in its original host *H. annosum* 94233 during *de novo* contig assembly of poly-A selected reads among which it was represented in very low quantity (library SRP097618; Vainio et al., 2019), and had been transmitted to 03021 during prior experiments (Kashif et al. 2019).

### 3.2 Effect of heat treatments

In order to obtain virus-free recipients, an attempt to cure ten *H. annosum* strains of the pool Elina01 was conducted using heat treatments, and the results are presented in the Supplementary Table 1. Suomusjärvi 1.4, Kortesjärvi 2.3.31, Hausjärvi 4.2 and Köyliö 6.21 were efficiently cured of HetOlV4-an1. Likewise, Suomusjärvi 1.4, Kortesjärvi 2.3.31 and Köyliö 6.21 were cured of curvulavirus HetRV6, but the heat-treatment was not successful for curing isolate Hausjärvi 11.8 of HetRV6-an8. Isolate Suomusjärvi 5.5 hosted nine different viruses before heat treatment, and it was cured of ambiviruses HetAlV14-an1, HetAlV15-an1, HetAlV16-an1 and ourmiavirus HetOlV4-an1, but not of partitiviruses HetPV13-an1, HetPV7-an3, HetPV3-an1, HetPV23-an1 and HetPV24-an1, and was discarded from transmission experiments. Transmission results of Suomusjärvi 5.5 were not analyzed, as it hosted HetPV13-an1.

### 3.3 Virus transmission frequencies

The results of the first transmission experiment (Table 1) indicate a high rate of transmission for HetPV13-an1, with consistent success across all recipient strains, particularly in the case of virus-free recipient strains. HetPV15-pa1 and HetOlV4-an1 displayed more variability in transmission success, as seen with the multiple virus infected strains S49-5 and 02018. In case of S49-5 the viruses HetPV15-pa1 and HetOlV4-an1 were transmitted poorly together. Instead, HetPV13-an1 and HetOlV4-an1 were transmitted successfully together in most replicates of S49-5. The opposite situation was observed with strain 02018 where HetPV13-an1 was transmitted in all five replicates, but HetOlV4-an1 in none. Recipients 02018 and S49-5 had earlier virus infection of HetOlV5, which might have affected the transmission rates, even though the identity of HetOlV4-an1 and HetOlV5 (MAG sequences) was relatively low (appr. 32 % global nt sequence alignment). We did not detect any mitovirus transmission in experiment 1.

**Table 1:** Results of the transmission experiment 1. The numbers of successful virus transmissions out of five replicates from donors 94233-PV13-MV1-2-OlV4 and 94233-PV15-MV1-2-3-OlV4 to six not heat-treated recipients are indicated. Abbreviations of virus names: PV = partitivirus; MV = mitovirus; OlV = ourmia-like virus, AlV = ambi-like virus, RV = RNA virus, NlV = narna-like virus. (The table notes *, ∅, #, ∈ are cited in Table body part).

⁎ Significant (*p* ≤ 0.05) differences in number of successful transmissions. Similar letter after star represents two compared transmission frequency columns.

∅ Recipient S49-5 contained HetOlV5-an2, HetAlV15-an3 and HetAlV16-an3 before transmission.

# Recipient 02018 contained HetRV6-an11, HetNlV3, HetOlV5-an1, HetAlV14-an2, HetAlV16-an2 and HetAlV17-an1 before transmission.

∈ Recipient KA 6.32A contained HetAlV15-an2 before transmission.

In the second experiment (Table 2) the transmission frequency was generally high from both donors, 03021-PV13-OlV4 and 03021-PV13-15-OlV4, to virus-free recipients. As an exception, the transmission of HetPV15-pa1 to KA 11.42A and 06066 from donor 03021-PV13-15-OlV4 was significantly lower than that of HetPV13-an1. The transmission success was variable to virus-hosting recipients. HetOlV4-an1 did not transmit from donor 03021-PV13-15-OlV4 to recipient 02018 and was thus significantly lower than transmission of HetPV13-an1. In addition, it should be mentioned that virus transmission frequencies in the recipient KA 6.32A seemed to be higher with the 94233 donors in the first experiment (Table 1) than what was seen with the 03021 donors (Table 2).

**Table 2:** Results of the transmission experiment 2. The numbers of successful virus transmissions out of five replicates from donors 03021-PV13-OlV4 and 03021-PV13-15-OlV4 to six not heat-treated recipients. Abbreviations of virus names: PV = partitivirus; MV = mitovirus; OlV = ourmia-like virus, AlV = ambi-like virus and RV = RNA virus. (The table notes *, ∅, #, ∈△ are cited in Table body part).

⁎ Significant (*p* ≤ 0.05) differences in number of successful transmissions. Similar letter after star represents two compared transmission frequency columns.

∅ Recipient S49-5 contained HetOlV5-an2, HetAlV15-an3 and HetAlV16-an3 before transmission.

# Recipient 02018 contained HetRV6-an11, HetNlV3, HetOlV5-an1, HetAlV14-an2, HetAlV16-an2 and HetAlV17-an1 before transmission.

∈ Recipient KA 6.32A contained HetAlV15-an2 before transmission.

△ Transmission frequency of at least one partitivirus, HetPV13-an1 or HetPV15-pa1.

In the third experiment the transmission frequencies of HetPV13-an1 and HetPV15-pa1 showed no significant differences between single and double partitivirus-infected donors in most recipients (Table 3). However, in the case of Suomusjärvi 1.4, HetPV13-an1 exhibited a high transmission frequency from single partitivirus-infected donor compared to double partitivirus-infected donor from which no transmission occurred. Additionally, both mitoviruses HetMV1-an1 and HetMV2-an1 were consistently transmitted to Köyliö K2 from 94233-PV13-MV1-2 across all five recipient replicates, whereas other mitovirus transmission was not observed. As Köyliö K2 was the only recipient where transmission of mitoviruses was observed, we conducted additional host purification (by picking the mycelium thrice from the edge of the mycelium) and RAMS profiling to exclude the possibility of contaminating donor hyphae being present in the recipient culture. The extra isolation step was successful from dual cultures of Köyliö K2 and the donor 94233-PV13-MV1-2-OlV4 (genotyping results reported in Supplementary Figure 1), but the recipient strain could not be re-isolated from the plate after dual culture with donors 94233-PV13-15-MV1-2-3-OlV4 and 94233-PV15-MV1-2-3-OlV4.

**Table 3:** Results of the transmission experiment 3. The numbers of successful virus transmissions out of five replicates from donors 94233-PV13-MV1-2-OlV4, 94233-PV15-MV1-2-3-OlV4 and 94233-PV13-15-MV1-2-3-OlV4 to seven heat-treated recipients. Abbreviations of virus names: PV = partitivirus; MV = mitovirus; OlV = ourmia-likevirus, RV = RNA-virus. (The table notes *,∅, △ are cited in Table body part).

⁎ Significant (*p* ≤ 0.05) differences in number of successful transmissions. Similar letter after star represents two compared transmission frequency columns.

∅ Recipient Hausjärvi 11.8 contained HetRV6-an8 before transmission.

△ Transmission frequency of at least one partitivirus, HetPV13-an1 or HetPV15-pa1. NA Not analyzed due to technical difficulties.

## 4 Discussion

Understanding factors that affect the transmission efficacy of viruses that cause host debilitation is essential for bringing virocontrol solutions closer to practice. In previous experiments partitiviruses HetPV13-an1 and HetPV15-pa1 were found to enhance each other´s transmission rates between *H. annosum* isolates (Kashif et al., 2019). To test whether this phenomenon is generalizable under laboratory conditions, we investigated a more diverse collection of *H. annosum* isolates in the present work, whereas Kashif et al. (2024) examined the same objective using *H. parviporum* strains. Our results with *H. annosum* showed that the relationship between double-infecting partitiviruses was considerably more complicated than expected. Thus, among the isolates tested in this study, transmission frequency of HetPV13-an1 and HetPV15-pa1 was not higher from double partitivirus-infected donors compared to single partitivirus-infected donors. This means that double partitivirus-infection is not required for efficient transmission of aforesaid viruses, and both HetPV13-an1 and HetPV15-pa1 have potential to be used separately or together in biocontrol. Further studies should examine if same result applies to field conditions, although Piri et al. (2023) observed transmission of HetPV13-an1 also *in vivo*. ssRNA viruses, such as mito- and ourmiaviruses were recently found to constitute an inherent part of the *H. annosum* (Dálya et al., 2024) and *H. parviporum* (Sutela et al., 2021) virome and are also present in the donor isolates of this study. This presented us a valuable opportunity to examine for the first time their transmission concomitantly with other viruses. We showed that transmission of HetOlV4-an1 was successful from double infected donor both alone and together with HetPV13-an1. This estimation of *in vitro* transmission frequency of ourmiaviruses in *H. annosum* complements the findings of Sutela et al. (2021) who found that many *H. parviporum* strains hosted very closely related HetOlV1-pa5 variants in a single clone and supports the recent finding of ourmiavirus transmission among *H. annosum* strains in Czechia (Dálya et al., 2024).

Based on our results, mitovirus transmission seems to be a rare event, as observed in only one donor-recipient pair (94233-PV13-MV1-2-OlV4 and Köyliö K2). Also, the donor strain from where the two mitoviruses were transmitted from was devoid of HetMV3, which may have affected the transmissions. Transmission of mitoviruses has been earlier shown in other fungal species like *Nigrospora oryzae* (Liu et al., 2019) and *Cryphonectria parasitica* (Suzuki et al., 2021). Notably, the mitochondrial haplotypes were not investigated here and there's a possibility that whole mitocondria could have been transmitted between strains (Suzuki et al., 2021).

This study was motivated by the fact that the presence of mycoviruses is common among isolates of *Heterobasidion* sp. (Vainio and Hantula, 2016; Sutela et al., 2021; Dálya et al., 2024), and therefore it is very likely that they would be present in indigenous *H. annosum* isolates when using a virocontrol preparation in field conditions. Therefore, it is essential to test whether their presence in recipients could hinder the transmission of viruses aimed to be used as biocontrol agents. Our results suggested that recipients hosting viruses infected by potential biocontrol agents HetPV13-an1 and HetPV15-pa1 were infected, but less frequently, than virus free ones. This observation calls for further research and accords with a finding that the presence of the mycoreovirus RnMyRV3 in the ascomycetous root rot fungus *Rosellinia necatrix* restricts the transmission of the partitivirus RnPV1 to the same host strain (Yaegashi et al., 2011). Possible underlying mechanism is cross-protection via RNA silencing which was previously studied with Mycoreovirus 1 that inhibited transcription of Rosellinia necatrix victorivirus 1 (Chiba and Suzuki, 2015).

As a conclusion, the transmission rates of the two partitiviruses were very high under laboratory conditions in *H. annosum,* but pre-existing virus infections or co-infecting viruses might lower the transmission frequency. Future studies should investigate the transmission of *H. annosum* mycoviruses using isogenic strains regarding the extant virus infections, as some viruses might suppress the vegetative incompatibility reaction and thereby facilitate virus transmission as seen with Sclerotinia sclerotiorum mycoreovirus 4 (Wu et al., 2017). Furthermore, some mycoviruses have RNA silencing suppressors, that can facilitate the establishment of the virus infections, leading to improved virus transmission efficacy (Yaegashi et al., 2013; Aulia et al., 2021). Ultimately, more field experiments are needed to understand mycovirus transmission in the root system, as also plant derived factors might interfere with the virus transmission. This was recently shown in *Brassica napa*, as mycovirus transmission between *Sclerotinia sclerotiorum* strains was enhanced *in planta* due to elevated proline concentration resulting from stress caused by the pathogen (Hai et al., 2024).

## Funding

This study was financially supported by The Finnish Society of Forest Science (E.R), University of Helsinki Doctoral Programme in Sustainable Use of Renewable Natural Resources (E.R) and the Academy of Finland (decision number 322001) (J.H).

## Author statement

I certify that all authors have read and approved the final version of the manuscript and contributed significantly to the work. The manuscript has not been published previously and is not being considered for publication elsewhere.

## Declaration of generative AI in scientific writing

During the preparation of this work the authors used OpenAI / ChatGPT in order to evaluate phrasing and grammar. After using this tool/service, the author(s) reviewed and edited the content as needed and takes full responsibility for the content of the publication.

## CRediT authorship contribution statement

**Elina Roininen:** Writing – review & editing, Writing – original draft, Supervision, Methodology, Investigation, Funding acquisition. **Eeva Johanna Vainio:** Writing – original draft, Supervision, Investigation, Funding acquisition. **Suvi Sutela:** Writing – review & editing, Writing – original draft, Supervision, Investigation. **Anna Poimala:** Writing – review & editing, Writing – original draft, Investigation. **Muhammad Kashif:** Writing – review & editing, Writing – original draft, Investigation, Funding acquisition. **Tuula Piri:** Writing – review & editing, Methodology, Investigation, Funding acquisition. **Jarkko Hantula:** Writing – review & editing, Writing – original draft, Supervision, Methodology, Investigation, Funding acquisition.

## Declaration of competing interest

The authors declare that they have no known competing financial interests or personal relationships that could have appeared to influence the work reported in this paper. Elina Roininen reports financial support was provided by The Finnish Society of Forest Science. Jarkko Hantula reports financial support was provided by Academy of Finland. If there are other authors, they declare that they have no known competing financial interests or personal relationships that could have appeared to influence the work reported in this paper.

## Data Availability

Data will be made available on request.

## Appendix Supplementary materials

Image, application 1
