# Supplementary material for: Virus transmission frequencies in the pine root rot pathogen Heterobasidion annosum
Source: Virus Res. 2024 Oct 7;350:199467. doi: 10.1016/j.virusres.2024.199467 (PMC11736397; doi:10.1016/j.virusres.2024.199467)
Supplement: Supplementary file 1 [file mmc1.docx]

**Supplementary material**

**Virus transmission frequencies of the pine root rot pathogen *Heterobasidion annosum***

1. **Supplementary Tables**

**Supplementary Table 1.** *H. annosum* strains of the study.

| **Strain name** | **RNA-Seq library or reference**^3)^ | **Heat treated** | **Viruses of the isolate**  **in the RNAseq** ^1)^ | **Viruses removed by**  **the heat treatment** | **Viruses after**  **the heat treatment** | **Use in this study** | **Origin information or reference**^4)^ |
| --- | --- | --- | --- | --- | --- | --- | --- |
| Lokalahti 3.9 | Elina01 | yes |  |  |  | Recipient, experiment 3 | Lokalahti, 2016, TP |
| Suomusjärvi 1.4^1)^ | “ | “ | HetRV6-an7, HetOlV4-an1 | HetRV6-an7, HetOlV4-an1 | - | “ | Suomusjärvi, 2016, TP |
| Kortesjärvi 1.1.6.5 | “ | “ | - |  |  | “ | Kortesjärvi, 2018, TP |
| Hausjärvi 11.8 | “ | “ | HetRV6-an8 | - | HetRV6-an8 | “ | Hausjärvi 11.8, 2018, TP |
| Kortesjärvi 2.3.31^1)^ | “ | “ | HetRV6-an10, HetOlV4-an1 | HetRV6-an10, HetOlV4-an1 | - | “ | Kortesjärvi, 2018, TP |
| Köyliö K2 | “ | “ | - |  |  | “ | Köyliö, 2018, TP |
| Köyliö 6.21 | “ | “ | HetRV6-an9, HetAlV16-an2 | HetRV6-an9, HetAlV16-an2 | - | “ | Köyliö, 2018, TP |
| Hausjärvi 4.2^1)^ | “ | “ | HetOlV4-an1 | HetOlV4-an1 | - | Not used in transmissions | Hausjärvi, 2018, TP |
| Lokalahti 1.7 | “ | “ | - |  |  | “ | Lokalahti 2016, TP |
| Suomusjärvi 5.5^1)^ | “ | “ | HetPV13-an1, HetPV7-an3, HetPV3-an1, HetPV23-an1, HetPV24-an1, HetOlV4-an1, HetAlV14-an1, HetAlV15-an1, HetAlV16-an1 | HetOlV4-an1, HetAlV14-an1, HetAlV15-an1, HetAlV16-an1 | HetPV13-an1, HetPV7-an3, HetPV3-an1, HetPV23-an1, HetPV24-an1 | “ | Suomusjärvi 2016, TP |
| KA 6.32A | Elina02 | no | HetAlV15-an2 |  |  | Recipient, experiments 1 & 2 | Karkkila 2007, TP |
| 06066 | “ | “ | - |  |  | “ | Alajärvi 2006, AP |
| 02018 | “ | “ | HetRV6-an11, HetNlV3, HetOlV5-an1, HetAlV14-an2, HetAlV16-an3, HetAlV17-an1 |  |  | “ | Toijala, 2002, KL & JA |
| S49-5 | “ | “ | HetOlV5-an2, HetAlV15-an3, HetAlV16-an4 |  |  | “ | Loppi, 2005  Hyder et al., 2018 |
| KA 401-10-58 | “ | “ | HetAlV17-an2 |  |  | “ | Karkkila 2007, TP |
| KA 11.42A | “ | “ | - |  |  | “ | Karkkila 2007, TP |
| 03021-PV13-OlV4^1)^ | “ | “ | HetPV13-an1, HetOlV4-an1 |  |  | Donor, experiment 2 | Kashif et al., 2019 |
| 03021-PV13-15-OlV4^1,2)^ |  | “ | HetPV13-an1, HetPV15-pa1, HetOlV4-an1 |  |  | Donor, experiment 2 | Kashif et al., 2019 |
| 94233-PV13-MV1-2-OlV4^1)^ | Vainio, 2019 | “ | HetPV13-an1, HetMV1, HetMV2, HetOlV4-an1 |  |  | Donor, experiment 1 & 3 | Krucz, 1994, PL |
| 94233-PV15-MV1-2-3-OlV4^1)^ | “ | “ | HetPV15-pa1, HetMV1, HetMV2, HetMV3, HetOlV4-an1 |  |  | “ | Kashif et al., 2019 |
| 94233-PV13-15-MV1-2-3-OlV4^1)^ | “ | “ | HetPV13-an1, HetPV15-pa1, HetMV1, HetMV2, HetMV3, HetOlV4-an1 |  |  | Donor, experiment 3 | Kashif et al., 2019 |

1. The principle of naming virus-hosting strains follows that of Hantula et al. (2020). HetPV=Heterobasidion partitivirus, HetOlV=Heterobasidion ourmia-like virus, HetAlV= Heterobasidion ambi-like virus, HetRV=Heterobasidion RNA virus, HetNlV= Heterobasidion narna-like virus. Virus variant indication is added to the end of the virus name.
2. *H. annosum* strain 94233 is probably the original host of HetOlV4-an1. Suomusjärvi 1.4^,^ Kortesjärvi 2.3.31, Suomusjärvi 5.5, Hausjärvi 4.2 and 03021 have obtained it during earlier experiments (Kashif et al. 2019, Piri et al. 2023), although some single point mutations have occurred thereafter.
3. Construct of strain 03021-PV13-OlV4 (H.a) hosting HetPV15-pa1. Not included in RNA-Seq separately.
4. Library Elina02 contained also one strain of each: *Lactarius rufus, Heterobasidion abietinum, Paxillus* sp.
5. TP, Tuula Piri; AP, Antti Pajula; KL, Katriina Lipponen; JA, J. Aarnio; PL, P. Lakomy

**Supplementary Table 2.** Primers used in the current study.

| **Target amplicon** | **Forward primer** | **Sequence of forward primer** | **Reverse primer** | **Sequence of reverse primer** | **Reference of primer** |
| --- | --- | --- | --- | --- | --- |
| CT (random  amplified microsatellite) | CT-primer | VDV(CT)7C | - | - | Hantula et al. (1996) |
| HetPV13-an1 | PV13midFor2 | TGCTCCTCTCCCGACTCTAC | PV13midR | CTTGGGTAGCCATTGTCGTT | Kashif et al. (2015) |
| HetPV15-pa1 | PV15midFor2 | TTGGGGATTCGAAACAATTC | PV15midR | CGAGCGATGTGATCGAAGTA | ” |
| HetMV1 | HetMV1MidF | CCTATGGATGGGACTTTTGACC | HetMV1MidRev | TTTGCGAATTCAAGCCCGAT | Vainio et al. (2015a) |
| HetMV2 | 2ndCONF1 | CGCTATCTTCGATTACTGATCACA | 2ndCONRev2 | GCTATACGCTCCCATTGGTTG | Vainio et al. (2015a) |
| HetMV3 | MV346 F | CAGGAATGGATAATAGCTTCG | 3rdMVMidR1 | AACATTTCCGCATCCGTTAG | Vainio (2019) |
| HetOlV4-an1 | SPOuConF2 | CCCRCGTAAAGTACCAGTGAAA | SPOuConRev2 | TCCCGGCACGAATACCA |  |
| HetOlV5-an1, HetOlV5-an2 | EDN560OVF1 | CTCGACCTACTTCCGCTCAC | EDN560OVR1 | GAAAAGGGAACCCGAAAGAG |  |
| HetAlV16-an3, HetAlV16-an4 | E02-DN1570-ambi9-F | ACCGTGACCAGGATCTTCAC | E02-DN1570-ambi9-R | CGAAAGACAGTGGAGCACAA |  |
| HetAlV16-an1, HetAlV16-an2 | EDN1514For1 | CACTACACTCCTCCACGGACT | EDN1514Rev1 | CCAACATTTCCTGGTTTACG |  |
| HetAlV15-an2, HetAlV15-an3 | E02-DN1571-ambi10-F | GAAGGAGAGGGAAGGTGGTC | E02-DN1571-ambi10-R | GCAGAATGTCGCGACTGTAA |  |
| HetRV6-an7, HetRV6-an9, HetRV6-an10, HetRV6-an11 | HV6F1A | TTGAATCACCTGGACCGTTT | HV6Re2 | CATCAACCCATTATCCAGGT | Vainio et al. (2012) |
| HetRV6-an8 | KPCurF1 | GTTTCAACCGCACCTACACC | HV6Re2 | CATCAACCCATTATCCAGGT |  |
| HetPV7-an3 | NPFor | ATGTCAACCAACCCTCCT | NPRev431 | TCGACGGGTGTAACTTCTTG | Vainio et al. (2015b) |
| HetPV3-an1 | DN32005_for1 | AATGGACAGGTCTCTCTGATCC | DN32005_rev1 | TGGTATGTAGGCAGTGAAATCG |  |
| HetPV23-an1 | DN7685PV7RPF1 | CTCGACATGAGTCCAAGCAA | DN7685PV7RPR1 | GAGGGCGTTCTGAGTAGTCG |  |
| HetPV23-an1 | EDN13538PV7CPF1 | TGTCTCAGCTTCCTCCTGGT | EDN13538PV7CPR1 | GGGAACTTATTGAGCGTGGA |  |
| HetPV24-an1 | DN30250_for1 | CCGATCTCATTACAATGCTGAA | DN30250_rev1 | ATCGAATCCAGACCAGTCAAGT |  |
| HetPV24-an1 | DN16663_for | ACGTCCTCCGCCTTTATTTT | DN16663_rev | CGTTCTCAGGAGACCTTTCG |  |
| HetAlV14-an1 | EDN7086For1 | AGCGAGAGATGCACCCTAAAG | EDN7086Rev1 | GCGCATCATTCTATCACGAAG |  |
| HetAlV14-an2 | E02-DN10942-ambi7-F | CAAGAGGGTGGTCAATTCGT | E02-DN10942-ambi7-R | TGAAACGGAATTGGAGAAGG |  |
| HetAlV15-an1 | EDN17576For1 | CCATCTGGTCCTCGTCATCT | EDN17576Rev1 | CTATCGCCTCAATCGGAAGA |  |
| HetNalV3-an1 | EDN54042NVF1 | TGACGCACTTCTCCAGATTG | EDN54042NVR1 | TTCTCTTCCCCATCATCCAG |  |
| HetAlV17-an1, HetAlV17-an2 | E02-DN4618-ambi8-F | CCATGGAAGGGATACCAATG | E02-DN4618-ambi8-R | CACCCTCTACCTTGCTACGC |  |

**Supplementary table 3.** Viruses detected in the RNA sequencing.

| **Virus** | **Infected**  ***H. annosum* strain/s** | **GenBank accession number of the virus** | **Type of GenBank sequence ^1)^** | **PCR target** | **Contig name** | **RNAseq library in Bioproject PRJNA1105565 ^3)^** | **Contig length (bp)** | **Map-**  **ping reads (thou-sands)** | **Mean cove-rage** | **Num-ber of SNPs** |
| --- | --- | --- | --- | --- | --- | --- | --- | --- | --- | --- |
| HetPV13-an1 | Suomusjärvi 5.5 | Kashif et al. (2015) |  | RdRp | TRINITY_DN560_c0_g1_i6 | Elina01 | 2249 | 36,711 | 1706.3 | 4 |
| HetPV13-an1 | Suomusjärvi 5.5 | ” |  | CP | TRINITY_DN22184_c0_g1_i1 | Elina01 | 1704 | 1,773 | 105.0 | 0 |
| HetPV13-an1 | 03021 | ” |  | RdRp | TRINITY_DN50748_c0_g1_i1 | Elina02 | 1840 | 1,677 | 91.2 | 0 |
| HetPV13-an1 | 03021 | ” |  | CP | TRINITY_DN24300_c0_g1_i1 | Elina02 | 1006 | 97 | 9.7 | 1 |
| HetOlV4-an1 | 03021 | OR343711 | MAG | RdRp | TRINITY_DN20053_c0_g1_i1 | Elina02 | 2636 | 3,577 | 135.9 | 0 |
| HetOlV4-an1 | Suomusjärvi 1.4, Suomusjärvi 5.5, Kortesjärvi 2.3.31, Hausjärvi 4.2 | Former trans-missions ^2)^ |  | RdRp | TRINITY_DN3243_c0_g1_i1 | Elina01 | 2645 | 70,288 | 2618.9 | 7 |
| HetOlV5-an1 | 02018 | OR343712 | Sanger | RdRp | TRINITY_DN560_c1_g1_i2 | Elina02 | 2856 | 6,288 | 215.8 | 57 |
| HetOlV5-an2 | S49-5 | OR343713 | Sanger | RdRp | " | Elina02 | " | " | " | " |
| HetAlV16-an3 | 02018 | OR343714 | Sanger | hypothetical protein | TRINITY_DN1570_c0_g1_i3 | Elina02 | 5442 | 390,709 | 7159.2 | 47 |
| HetAlV16-an4 | S49-5 | OR343715 | Sanger | hypothetical protein | " | Elina02 | " | " | " | " |
| HetAlV16-an1 | Suomusjärvi 5.5 | OR343716 | Sanger | intergenic region | TRINITY_DN1514_c0_g1_i5 | Elina01 | 5566 | 461,081 | 8202.8 | 210 |
| HetAlV16-an2 | Köyliö 6.21 | OR343717 | Sanger | intergenic region | " | Elina01 | " | " | " | " |
| HetAlV15-an2 | KA 6.21 | OR343718 | Sanger | hypothetical protein | TRINITY_DN1571_c0_g1_i1 | Elina02 | 5368 | 16,081 | 296.2 | 141 |
| HetAlV15-an3 | S49-5 | OR343719 | Sanger | hypothetical protein | " | Elina02 | " | " | " | " |
| HetRV6-an8 | Hausjärvi 11.8 | OR343721 | Sanger | RdRp | TRINITY_DN4991_c1_g1_i2 | Elina01 | 1959 | 1,07 | 55 | 73 |
| HetRV6-an7 | Suomusjärvi 1.4 | OR343720 | Sanger | RdRp | " | Elina01 | " | " | " | " |
| HetRV6-an9 | Köyliö 6.21 | OR343722 | Sanger | RdRp | " | Elina01 | " | " | " | " |
| HetRV6-an10 | Kortesjärvi 2.3.31 | OR343723 | Sanger | RdRp | " | Elina01 | " | " | " | " |
| HetRV6-an11 | 02018 | OR343724 | Sanger | RdRp | TRINITY_DN8581_c0_g2_i1 | Elina02 | 1975 | 658 | 32.8 | 110 ^4)^ |
| HetPV7-an3 | Suomusjärvi 5.5 | OR343725 | MAG | RdRp | TRINITY_DN17503_c0_g1_i1 | Elina01 | 2251 | 2,777 | 124.6 | 0 |
| HetPV7-an3 | Suomusjärvi 5.5 | OR343726 | MAG | Cp | TRINITY_DN28099_c0_g1_i1 | Elina01 | 2186 | 2,564 | 117.0 | 2 |
| HetPV3-an1 | Suomusjärvi 5.5 | OR343727 | MAG | RdRp | TRINITY_DN32005_c0_g1_i1 | Elina01 | 1854 | 4,605 | 250.7 | 3 |
| HetPV3-an1 | Suomusjärvi 5.5 | OR343728 | MAG | Cp | TRINITY_DN29439_c0_g1_i1 | Elina01 | 1764 | 2,714 | 154.8 | 0 |
| HetPV23-an1 | Suomusjärvi 5.5 | OR343729 | MAG | RdRp | TRINITY_DN7685_c0_g1_i1 | Elina01 | 2264 | 10,063 | 445.2 | 0 |
| HetPV23-an1 | Suomusjärvi 5.5 | OR343730 | MAG | Cp | TRINITY_DN13538_c0_g2_i1 | Elina01 | 2137 | 779 | 36 | 3 |
| HetPV24-an1 | Suomusjärvi 5.5 | OR343731 | MAG | RdRp | TRINITY_DN30250_c0_g1_i1 | Elina01 | 1981 | 37,138 | 1888.5 | 1 |
| HetPV24-an1 | Suomusjärvi 5.5 | OR343732 | MAG | Cp | TRINITY_DN16663_c2_g1_i1 | Elina01 | 1420 | 610 | 43,5 | 0 |
| HetAlV14-an1 | Suomusjärvi 5.5 | OR343733 | Sanger | hypothetical protein | TRINITY_DN7086_c0_g1_i1 | Elina01 | 4910 | 12,776 | 256.8 | 0 |
| HetAlV14-an2 | 02018 | OR343734 | Sanger | hypothetical protein | TRINITY_DN10942_c0_g1_i4 | Elina02 | 4923 | 6,186 | 124.8 | 24 |
| HetAlV15-an1 | Suomusjärvi 5.5 | OR343735 | MAG | hypothetical protein | TRINITY_DN14464_c0_g1_i1 | Elina01 | 4924 | 8,43 | 167.7 | 4 |
| HetNalV3-an1 | 02018 | OR343736 | MAG | hypothetical protein | TRINITY_DN54042_c0_g1_i1 | Elina02 | 3878 | 437,002 | 11017.1 | 0 |
| HetAlV17-an1 | 02018 | OR343737 | Sanger | hypothetical protein | TRINITY_DN4618_c0_g1_i3 | Elina02 | 5239 | 16,819 | 319.5 | 40 |
| HetAlV17-an2 | KA 401-10-58 | OR343738 | Sanger | hypothetical protein | " | Elina02 | " | " | " | " |

1. Metagenome Assembled Genome (MAG), Sanger sequence (Sanger)
2. 94233 is probably the original host of HetOlV4-an1. Suomusjärvi 1.4^,^ Kortesjärvi 2.3.31, Suomusjärvi 5.5, Hausjärvi 4.2 and 03021 have obtained it during earlier experiments (Kashif et al. 2019, Piri et al. 2023), although some single point mutations have occurred thereafter.
3. Library Elina02 contained also one strain of each species: *Lactarius rufus, Heterobasidion abietinum, Paxillus sp*. RNAseq results of these strains are not included in the current study.
4. Another variant was found from *H. abietinum*.

**Supplementary table 4.** Relation of the detected viruses to known viruses, BlastX analyses.

| **Virus** | **Accession number** | **Length of accession (nt)** | **Name of nearest hit, BlastX** | **Accession number of BlastX hit** | **Query Cover** | **E value** | **Percent identity** |
| --- | --- | --- | --- | --- | --- | --- | --- |
| HetOlV4-an1 | OR343711 | 2597 | RNA-dependent RNA polymerase [Heterobasidion ourmia-like virus 3] | UOX39321.1 | 73 % | 0.0 | 94.37 |
| HetOlV5-an1 | OR343712 | 479 | RNA-dependent RNA polymerase [Heterobasidion ourmia-like virus 2] | UOX39320.1 | 51 % | 1,00E-28 | 95.18 |
| HetOlV5-an2 | OR343713 | 477 | RNA-dependent RNA polymerase [Heterobasidion ourmia-like virus 2] | UOX39320.1 | 52 % | 1,00E-28 | 95.18 |
| HetAlV16-an3 | OR343714 | 503 | hypothetical protein [Heterobasidion ambi-like virus 5] | UOX39305.1 | 99 % | 1,00E-105 | 98.80 |
| HetAlV16-an4 | OR343715 | 503 | hypothetical protein [Heterobasidion ambi-like virus 5] | UOX39305.1 | 99 % | 2,00E-106 | 100.00 |
| HetAlV16-an1 | OR343716 | 393 | hypothetical protein [Heterobasidion ambi-like virus 5] | UOX39306.1 | 99 % | 8,00E-81 | 95.38 |
| HetAlV16-an2 | OR343717 | 393 | hypothetical protein [Heterobasidion ambi-like virus 5] | UOX39306.1 | 99 % | 8,00E-80 | 94.62 |
| HetAlV15-an2 | OR343718 | 422 | hypothetical protein [Heterobasidion ambi-like virus 6] | UOX39307.1 | 99 % | 4,00E-92 | 98.57 |
| HetAlV15-an3 | OR343719 | 422 | hypothetical protein [Heterobasidion ambi-like virus 6] | UOX39307.1 | 99 % | 3,00E-89 | 95.71 |
| HetRV6-an8 | OR343721 | 621 | RNA-dependent RNA polymerase [Heterobasidion RNA virus 6] | AHL25164.1 | 99 % | 2,00E-150 | 99.51 |
| HetRV6-an7 | OR343720 | 621 | RNA-dependent RNA polymerase [Heterobasidion RNA virus 6] | AHL25164.1 | 99 % | 2,00E-150 | 99.51 |
| HetRV6-an9 | OR343722 | 621 | putative RNA-dependent RNA polymerase [Heterobasidion RNA virus 6] | ADW82841.1 | 99 % | 1,00E-150 | 100.00 |
| HetRV6-an10 | OR343723 | 621 | putative RNA-dependent RNA polymerase [Heterobasidion RNA virus 6] | ADW82841.1 | 99 % | 1,00E-150 | 100.00 |
| HetRV6-an11 | OR343724 | 621 | putative RNA-dependent RNA polymerase [Heterobasidion RNA virus 6] | ADW82841.1 | 99 % | 8,00E-150 | 99.51 |
| HetPV7-an3 | OR343725 | 2249 | RdRp protein [Heterobasidion partitivirus 7] | AST13828.1 | 96 % | 0.0 | 99.59 |
| HetPV7-an3 | OR343726 | 2186 | [capsid protein [Heterobasidion partitivirus 7]](https://blast.ncbi.nlm.nih.gov/Blast.cgi#alnHdr_YP_009408641) | YP_009408641.1 | 89 % | 0.0 | 99.69 |
| HetPV3-an1 | OR343727 | 1847 | putative RNA-dependent RNA polymerase [Heterobasidion partitivirus 3] | YP_009508058.1 | 94 % | 0.0 | 91.78 |
| HetPV3-an1 | OR343728 | 1760 | putative coat protein [Heterobasidion partitivirus 3] | YP_009508057.1 | 88 % | 0.0 | 80.27 |
| HetPV23-an1 | OR343729 | 2234 | RNA-dependent RNA polymerase [Heterobasidion partitivirus 7] | YP_009408640.1 | 97 % | 0.0 | 64.23 |
| HetPV23-an1 | OR343730 | 2137 | putative coat protein [Heterobasidion partitivirus 2] | YP_009508062.1 | 91 % | 6,00E-158 | 41.55 |
| HetPV24-an1 | OR343731 | 1881 | RNA-dependent RNA polymerase [Heterobasidion partitivirus 21] | UHK02580.1 | 94 % | 0.0 | 71.79 |
| HetPV24-an1 | OR343732 | 1256 | coat protein [Sclerotinia sclerotiorum alphapartitivirus 1] | WEQ50557.1 | 84 % | 4,00E-155 | 64.49 |
| HetAlV14-an1 | OR343733 | 4910 | hypothetical protein [Heterobasidion ambi-like virus 22] | WBE16496.1 | 42 % | 0.0 | 71.06 |
| HetAlV14-an2 | OR343734 | 4923 | hypothetical protein [Heterobasidion ambi-like virus 22] | WBE16496.1 | 42 % | 0.0 | 70.34 |
| HetAlV15-an1 | OR343735 | 5107 | hypothetical protein [Heterobasidion ambi-like virus 6] | UOX39308.1 | 43 % | 0.0 | 99.33 |
| HetNalV3-an1 | OR343736 | 3878 | RNA-dependent RNA polymerase [Heterobasidion narna-like virus 2] | UOX39318.1 | 94 % | 0.0 | 98.60 |
| HetAlV17-an1 | OR343737 | 359 | hypothetical protein [Heterobasidion ambi-like virus 9] | UOX39313.1 | 99 % | 2,00E-76 | 100.00 |
| HetAlV17-an2 | OR343738 | 359 | hypothetical protein [Heterobasidion ambi-like virus 20] | WBE16491.1 | 99 % | 4,00E-75 | 97.48 |

1. **Supplementary Figures**


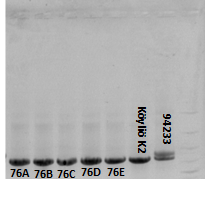


**Supplementary Figure 1.** RAMS genotyping of recipient Köyliö K2 transmission replicates and donor 94233-PV13-MV1-2.

**References**

Hantula, J., Dusabenyagasani, M., & Hamelin, R. C. (1996). Random amplified microsatellites (RAMS)—a novel method for characterizing genetic variation within fungi. *European Journal of Forest Pathology*, *26*(3), 159-166. <https://doi.org/10.1111/j.1439-0329.1996.tb00720.x>

Hantula, J., Mäkelä, S., Xu, P., Brusila, V., Nuorteva, H., Kashif, M., Hyder, R., & Vainio, E. J. (2020). Multiple virus infections on *Heterobasidion* sp. *Fungal Biology, 124*(2). <https://doi.org/10.1016/j.funbio.2019.12.004>

Kashif, M., Hyder, R., Perez, D. D. V., Hantula, J., & Vainio, E. J. (2015). Heterobasidion wood decay fungi host diverse and globally distributed viruses related to Helicobasidium mompa partitivirus V70. *Virus research, 195*, 119-123. <https://doi.org/10.1016/j.virusres.2014.09.002>

Kashif, M., Jurvansuu, J., Vainio, E. J., & Hantula, J. (2019). Alphapartitiviruses of Heterobasidion wood decay fungi affect each other's transmission and host growth. *Frontiers in Cellular and Infection Microbiology, 9*, 64. <https://doi.org/10.3389/fcimb.2019.00064>

Piri, T., Vainio, E. J., & Hantula, J. (2023). Preventing mycelial spread of *Heterobasidion annosum* in young Scots pine stands using fungal and viral biocontrol agents. *Biological Control*, *184*. <https://doi.org/10.1016/j.biocontrol.2023.105263>

Vainio, E. J., Hyder, R., Aday, G., Hansen, E., Piri, T., Doğmuş-Lehtijärvi, T., ... & Hantula, J. (2012). Population structure of a novel putative mycovirus infecting the conifer root-rot fungus *Heterobasidion annosum* sensu lato. *Virology*, *422*(2), 366-376. https://doi.org/10.1016/j.virol.2011.10.032

Vainio, E. J., Jurvansuu, J., Streng, J., Rajamäki, M. L., Hantula, J., & Valkonen, J. P. (2015a). Diagnosis and discovery of fungal viruses using deep sequencing of small RNAs. *Journal of general virology*, *96*(3), 714-725. <https://doi.org/10.1099/jgv.0.000003>

Vainio, E. J., Müller, M. M., Korhonen, K., Piri, T., & Hantula, J. (2015b). Viruses accumulate in aging infection centers of a fungal forest pathogen. *The ISME Journal*, *9*(2), 497-507. <https://doi.org/10.1038/ismej.2014.145>

Vainio, E. J. (2019). Mitoviruses in the conifer root rot pathogens *Heterobasidion annosum* and *H. parviporum*. *Virus research*, *271*, 197681. <https://doi.org/10.1016/j.virusres.2019.197681>
